# IL-1β/IL-1Ra ratio dysregulation in islet autoantibody–positive adult-onset diabetes

**DOI:** 10.3389/fimmu.2026.1784582

**Published:** 2026-04-10

**Authors:** Bibigul Tleumagambetova, Khatimya Kudabayeva, Yerlan Bazargaliyev, Lorina Vudu

**Affiliations:** 1 Department of Internal Diseases № 1, West Kazakhstan Marat Ospanov Medical University, Aktobe, Kazakhstan; 2 The Department of Endocrinology and the Endocrinology Laboratory Nicolae Testemitanu State University of Medicine and Pharmacy, Chisinau, Moldova

**Keywords:** IL-1 beta, IL-1Ra, islet autoantibodies, Kazakhstan, β-cell dysfunction

## Abstract

**Background:**

The IL-1 pathway plays a central role in β-cell dysfunction; however, the relationship between IL-1β/IL-1Ra imbalance and islet autoimmunity in newly diagnosed adult-onset diabetes remains insufficiently defined. This study aimed to evaluate whether dysregulation of the IL-1β/IL-1Ra ratio differs according to islet autoantibody status.

**Methods:**

This cross-sectional study included 240 adults with newly diagnosed adult-onset diabetes recruited from primary healthcare facilities. Serum IL-1β and IL-1Ra concentrations were measured using enzyme-linked immunosorbent assays, and the IL-1β/IL-1Ra ratio was calculated as an index of inflammatory regulation. Islet autoantibodies to glutamic acid decarboxylase (GADA), insulinoma-associated protein-2 (IA-2A), zinc transporter 8 (ZnT8A), and islet cells (ICA) were determined using standardized ELISA assays. Associations were evaluated using nonparametric tests, multivariate logistic regression, and receiver operating characteristic (ROC) analysis.

**Results:**

Circulating IL-1β concentrations did not differ between autoantibody-positive and autoantibody-negative individuals. In contrast, IL-1Ra levels were significantly lower in autoantibody-positive patients (p = 0.029). Consequently, the IL-1β/IL-1Ra ratio was significantly higher in autoantibody-positive individuals compared with autoantibody-negative patients (p = 0.030). Among autoantibody-positive participants, the IL-1β/IL-1Ra ratio was significantly higher in individuals with single autoantibody positivity compared to those with multiple autoantibodies (p = 0.018), indicating a non-linear relationship between autoimmune burden and inflammatory imbalance. In multivariate analysis, a higher IL-1β/IL-1Ra ratio (OR 1.94, 95% CI 1.10–3.44; p = 0.024) and lower basal C-peptide levels (OR 0.50, 95% CI 0.33–0.76; p = 0.001) were independently associated with autoantibody positivity. ROC analysis demonstrated modest but statistically significant discriminative performance of the IL-1β/IL-1Ra ratio (AUC = 0.59, p = 0.030).

**Conclusion:**

Dysregulation of the IL-1β/IL-1Ra ratio is evident at the time of diabetes diagnosis and varies according to islet autoantibody status. The observed non-linear pattern, with a higher ratio in single autoantibody positivity, suggests stage-dependent inflammatory regulation and supports the concept of immunometabolic heterogeneity in adult-onset diabetes. While the IL-1β/IL-1Ra ratio reflects early inflammatory imbalance, its clinical utility as a standalone biomarker appears limited.

## 1 Introduction

Type 2 diabetes mellitus (T2DM) is a heterogeneous disease based on a complex interaction between metabolic and immune processes, which were previously considered independent, but are now recognized as closely interconnected parts of a single regulatory system (1). T2DM develops gradually, from a latent prodromal period to overt disease with impaired glucose tolerance. Gradual depletion of β-cells is accompanied by inflammation, apoptosis, and fibrosis of pancreatic tissue (2). This progressive β-cell dysfunction and tissue inflammation suggest that immune-mediated mechanisms play a central role in the pathogenesis of T2DM. In recent years, growing evidence has shown that low-grade chronic inflammation is a key contributor to the development of insulin resistance and β-cell dysfunction (3).

Among the proinflammatory mediators involved in T2DM, interleukin-1 beta (IL-1β) occupies a central position due to its dual, dependent effects on pancreatic β-cells. Under physiological conditions, low concentrations of IL-1β have been shown to support β-cell function and insulin secretion, whereas chronic exposure induces inflammation-mediated β-cell dysfunction and apoptosis (4). However, clinical studies have reported inconsistent findings regarding circulating IL-1β levels, with meta-analyses demonstrating no significant differences between patients with type 2 diabetes and controls (5). This “double-edged sword” effect of IL-1β highlights its pivotal role in the transition from adaptive to pathological immune responses in diabetes (6).

In contrast, the interleukin-1 receptor antagonist (IL-1Ra) is an endogenous anti-inflammatory mediator that regulates glucose metabolism by blocking IL-1β binding to its receptor and protecting β-cells from cytokine-induced injury (7). Elevated IL-1Ra levels represent an early compensatory response to low-grade inflammation and may appear several years before the onset of T2DM (8). Therefore, dysregulation of the IL-1β/IL-1Ra balance may reflect chronic low-grade inflammation and serve as an early indicator of metabolic dysfunction and progression toward T2DM (9). Given the context-dependent effects of IL-1β and the compensatory nature of IL-1Ra, their relative balance rather than absolute concentrations may more accurately reflect immunoinflammatory dysregulation in early T2DM (4, 10).

Considering the growing evidence linking inflammation and autoimmunity in diabetes (11), the present study aimed to investigate whether dysregulation of the IL-1β/IL-1Ra ratio differs between autoantibody-positive and autoantibody-negative patients with respect to pancreatic β-cell autoantibodies to glutamic acid decarboxylase (GADA), insulinoma-associated protein 2/tyrosine phosphatase (IA-2), zinc transporter 8 (ZnT8), and islet cell autoantibodies (ICA), thereby clarifying the role of IL-1-mediated inflammation as a potential mechanistic link between autoimmune and non-autoimmune pathways in newly diagnosed adult-onset diabetes.

## 2 Materials and methods

### 2.1 Study design and participants

This study was designed as a cross-sectional observational analysis of adults with newly diagnosed adult-onset diabetes receiving outpatient endocrine care in the Aktobe region of Kazakhstan. A total of 240 patients aged 30–75 years were recruited from primary healthcare facilities between May 1, 2024, and January 4, 2025. Participants were enrolled using a consecutive inclusion approach, whereby all eligible patients presenting during the study period were assessed for participation until the predefined sample size was achieved. To minimise selection bias, only patients without prior insulin therapy, without clinical features suggestive of classical type 1 diabetes at presentation, and with a known duration of diabetes of less than 12 months and no prior use of glucose-lowering therapy beyond initial treatment were included. All participants provided informed consent prior to inclusion.

This study included adults with newly diagnosed diabetes identified in routine clinical practice who lacked classical clinical features of type 1 diabetes at presentation. Diabetes was diagnosed according to the glycaemic criteria of the American Diabetes Association (ADA), with no history of diabetic ketoacidosis or insulin treatment during the early course of the disease (12). After testing for pancreatic islet autoantibodies, participants were stratified according to autoantibody status. Individuals with at least one positive autoantibody were classified as autoantibody-positive diabetes, reflecting autoimmune involvement, whereas those negative for all tested autoantibodies were classified as autoantibody-negative diabetes.

### 2.2 Clinical and anthropometric assessment

All participants underwent standardized clinical and anthropometric evaluation using a structured case report form. Anthropometric measurements included height, weight, body mass index (BMI) (13).

### 2.3 Laboratory investigations

Venous blood samples were collected in the morning after an overnight fast using BD Vacutainer systems and processed according to standardized laboratory procedures. Serum samples were aliquoted and stored at −20 °C until analysis.

Glycaemic and metabolic parameters

Fasting plasma glucose and HbA1c were measured using automated biochemical analysers (Mindray BS-240 Pro). HbA1c was determined by latex-enhanced immunoassay and expressed according to NGSP standards (13).

Serum insulin and C-peptide (basal and stimulated) concentrations were measured by chemiluminescent immunoassays (Mindray and MAGLUMI platforms). Stimulated C-peptide was assessed using a standard 75-g oral glucose tolerance test, with sampling at 120 minutes.

### 2.4 Measurement of islet autoantibodies

Serum autoantibodies against pancreatic β-cell antigens were analysed to determine autoimmune involvement. GADA and ZnT8 autoantibodies were measured using quantitative ELISA kits (ImmunoDiagnostics Limited, Hong Kong). IA-2 and ICA were determined using commercial ELISA assays (ELK Biotech, China).

All assays were performed according to manufacturers’ instructions. Autoantibody positivity was defined using validated cut-off values provided by the manufacturers. Analytical performance characteristics were within accepted ranges reported by international diabetes autoantibody standardisation programmes (14). Patients were classified as autoantibody-positive if at least one autoantibody was detected and as autoantibody-negative if all tested autoantibodies were absent.

### 2.5 Measurement of inflammatory markers

Serum concentrations of IL-1β and IL-1Ra were measured using sandwich enzyme-linked immunosorbent assays (ELK Biotech, China). The assays demonstrated high analytical sensitivity and specificity with no significant cross-reactivity.

Samples were analysed in duplicate, and concentrations were calculated from standard calibration curves. The IL-1β/IL-1Ra ratio was calculated as an index of imbalance within the IL-1 axis.

### 2.6 Statistical analysis

Descriptive statistics were calculated for continuous variables and presented as medians with interquartile ranges or as frequencies and percentages for categorical variables. The normality of data distribution was assessed using the Shapiro-Wilk test and demonstrated non-normality for most continuous variables.

Accordingly, group differences were analysed with the Mann-Whitney U test for continuous variables. When comparing more than two groups, differences were assessed using the Kruskal-Wallis test with appropriate *post hoc* interpretation. Logistic regression analysis was applied to explore factors independently associated with pancreatic β-cell autoantibody positivity. ROC curve analysis was performed to evaluate the discriminatory performance of inflammatory and metabolic markers for pancreatic β-cell autoantibody positivity. AUC values with 95% confidence intervals were calculated. The optimal cut-off value was determined using the Youden index, and corresponding sensitivity, specificity, positive predictive value (PPV), and negative predictive value (NPV) were calculated.

Variables of clinical and biological relevance were first examined in univariate models, followed by multivariate logistic regression to identify independent predictors while accounting for potential confounders. A two-sided p-value <0.05 was considered statistically significant.

All statistical analyses were performed using appropriate statistical software: IBM SPSS Statistics for macOS, Version 26 (IBM Corp., Armonk, NY, USA) for descriptive analysis, normality testing, group comparisons and logistic regression. RStudio for macOS, Version 2023.12.1 + 402 (Posit Software, PBC, Boston, MA, USA) for data visualization and correlation plots, and GraphPad Prism for macOS, Version 9.5.0 (GraphPad Software, LLC, San Diego, CA, USA) for graphical presentation of data.

## 3 Results

### 3.1 Descriptive characteristics of the study population

A total of 240 patients with newly diagnosed adult-onset diabetes were included in the study. Baseline demographic, anthropometric, metabolic, immunological, and inflammatory characteristics of the study population are summarized in **Table 1**.

**Table 1:** Baseline demographic, anthropometric, metabolic, immunological, and inflammatory characteristics of the study population.

Variable	Median [IQR]
Age, years	50.0 [40.0–59.8]
Body mass index, kg/m²	29.1 [25.7–32.8]
HbA1c, %	7.93 [6.50–10.82]
Fasting glucose, mmol/L	7.92 [5.82–11.61]
Fasting insulin, IU/mL	12.2 [7.24–21.22]
Basal C-peptide, ng/mL	2.14 [1.28–3.25]
Stimulated C-peptide, ng/mL	2.11 [0.63–3.88]
ZnT8 autoantibodies, U/mL	2.56 [2.12–3.17]
GAD autoantibodies, U/mL	32.9 [23.3–50.2]
IA-2 autoantibodies, U/mL	271.0 [115.4–475.8]
ICA antibodies, ng/mL	4.93 [2.23–9.82]
IL-1β, pg/mL	42.5 [38.3–50.0]
IL-1Ra, pg/mL	132.2 [64.8–218.0]

### 3.2 Association of islet autoantibody status with IL-1β and IL-1Ra levels in newly diagnosed adult-onset diabetes

To explore the relationship between autoimmune markers and inflammatory activity, serum IL-1β and IL-1Ra levels were first analysed separately according to the presence of individual islet autoantibodies: GADA, ZnT8A, IA-2A, and ICA (**Table 2**).

**Table 2:** IL-1β and IL-1Ra levels according to islet autoantibody status.

Variable	Median [IQR]		Z	P-value
	GADA positive (n=24, 10%)	GADA negative (n=216, 90%)		
IL-1β (pg/mL)	41.9 [38.4-50.0]	42.7 [38.2-50.0]	−0.18	0.85
IL-1Ra (pg/mL)	152.0 [60.7-230.1]	131.0 [65.6-218.0]	−0.05	0.95
IL-1β/IL-1Ra ratio	0.30 [0.18–0.76]	0.30 [0.20–0.68]	−0.09	0.93
	ZnT8A positive (n=41, 17.1%)	ZnT8A negative (n=199, 82.9%)		
IL-1β (pg/mL)	41.8 [36.5-51.9]	42.9 [38.5-50.0]	−0.18	0.85
IL-1Ra (pg/mL)	91.3 [62.7-179.2]	146.2 [64.7-226.6]	−1.57	0.11
IL-1β/IL-1Ra ratio	0.44 [0.24–0.74]	0.30 [0.18–0.67]	−1.32	0.19
	IA-2A positive (n=5, 2.1%)	IA-2A negative (n=235, 97.9%)		
IL-1β (pg/mL)	44.5 [34.8-55.9]	42.5 [38.3-50.0]	−0.12	0.90
IL-1Ra (pg/mL)	69.1 [43.4-284.5]	132.4 [65.1-218.0]	−0.44	0.66
IL-1β/IL-1Ra ratio	0.55 [0.16–1.05]	0.30 [0.19–0.68]	−1.51	0.13
	ICA positive (n=5, 2.1%)	ICA negative (n=235, 97.9%)		
IL-1β (pg/mL)	38.6 [35.7-46.5]	42.6 [38.4-50.0]	−1.02	0.30
IL-1Ra (pg/mL)	176.0 [53.4-284.5]	131.9 [64.7-218.0]	−0.34	0.73
IL-1β/IL-1Ra ratio	0.20 [0.16–0.74]	0.31 [0.19–0.68]	−0.98	0.33

As no significant differences were identified when IL-1β and IL-1Ra concentrations were analysed according to individual islet autoantibody specificities, subsequent analyses were performed based on overall islet autoantibody status. Of the 240 patients included, 74.2% (95% CI: 69.3-80.2) were autoantibody-negative, whereas 25.8% (95% CI: 19.8-30.7) were autoantibody-positive.

**Figure 1:**
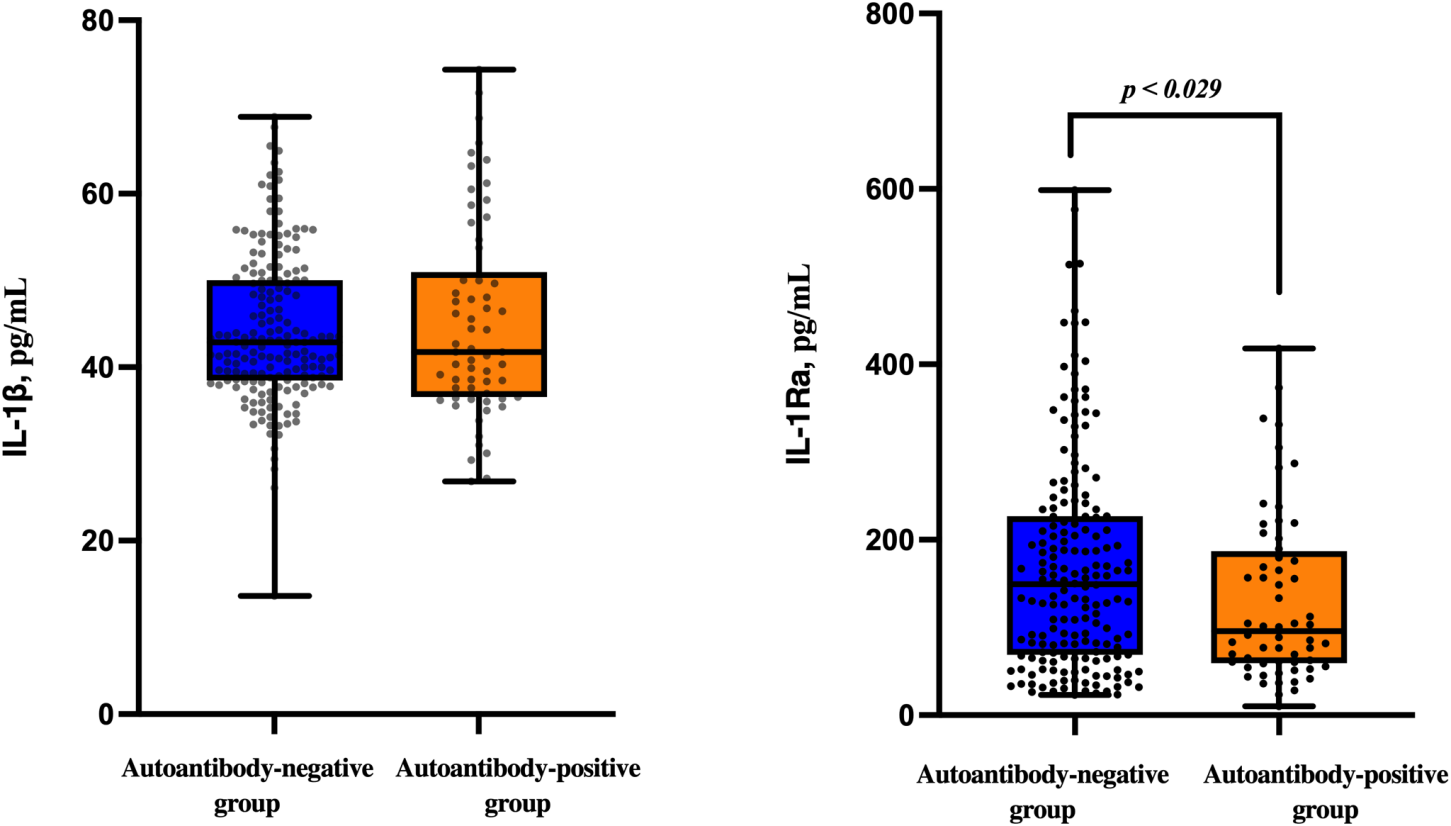
Comparison of serum IL-1β and IL-1Ra levels between autoantibody-negative (n=178) and autoantibody-positive (n=62) patients. Differences were assessed using the Mann–Whitney U test.

Serum IL-1β levels did not differ significantly between autoantibody-negative and autoantibody-positive patients. In contrast, IL-1Ra concentrations were significantly lower in autoantibody-positive patients compared with autoantibody-negative individuals (p = 0.029), suggesting reduced anti-inflammatory counter-regulation in the presence of islet autoimmunity (**Figure 1**).

Patients were further stratified by autoantibody burden into the autoantibody-negative, single autoantibody-positive, and multiple autoantibody-positive groups. Our results demonstrated a significant difference in IL-1Ra levels across groups (H = 9.934, p = 0.007).

**Figure 2:**
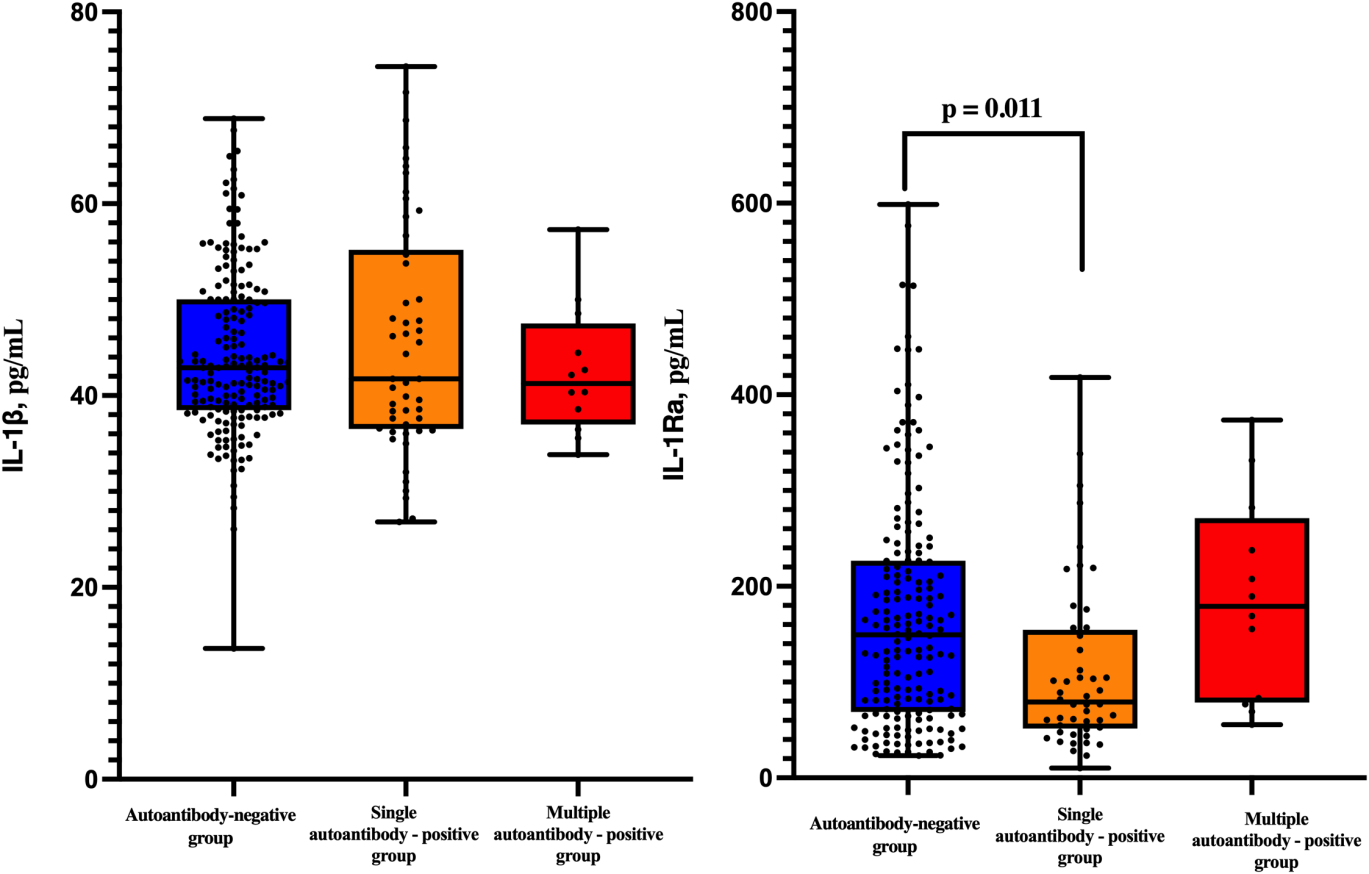
Comparison of serum IL-1β and IL-1Ra levels across autoantibody burden groups: islet autoantibody-negative (n=178), single autoantibody-positive (n=50), and multiple autoantibody-positive (n=12). Differences were assessed using the Kruskal–Wallis test with Dunn’s *post hoc* analysis.

*Post hoc* pairwise comparisons using Dunn’s test with Bonferroni correction revealed that IL-1Ra concentrations were significantly lower in patients positive for a single autoantibody compared with autoantibody-negative individuals (adjusted p = 0.011). No significant differences were observed between autoantibody-negative patients and those with multiple autoantibodies, nor between single- and multiple-autoantibody-positive patients (**Figure 2**).

### 3.3 Regulatory imbalance within the IL-1β/IL-1Ra ratio

To further characterise the balance between pro- and anti-inflammatory components of the IL-1 axis, the IL-1β/IL-1Ra ratio was calculated for each participant and analysed according to islet autoantibody status.

**Figure 3:**
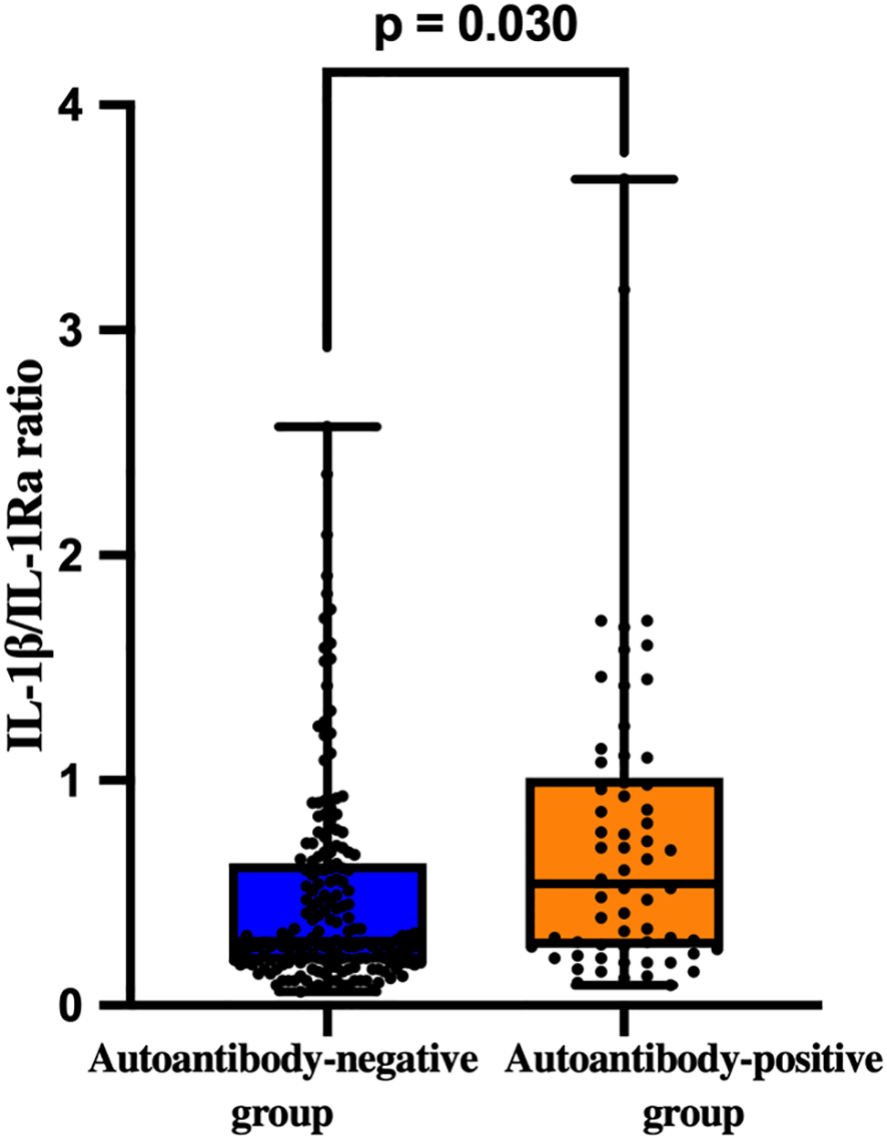
Comparison of the IL-1β/IL-1Ra ratio between autoantibody-negative (n=178) and autoantibody-positive (n=62) patients. Differences were assessed using the Mann–Whitney U test.

Overall, patients with newly diagnosed adult-onset diabetes demonstrated a marked interindividual variability in the IL-1β/IL-1Ra ratio, indicating heterogeneity of inflammatory regulation. When patients were stratified by overall islet autoantibody status, the IL-1β/IL-1Ra ratio was significantly higher in autoantibody-positive patients compared with autoantibody-negative individuals (Z = −2.164, p = 0.030), reflecting a relative predominance of pro-inflammatory signalling in the presence of autoimmune markers (**Figure 3**).

**Figure 4:**
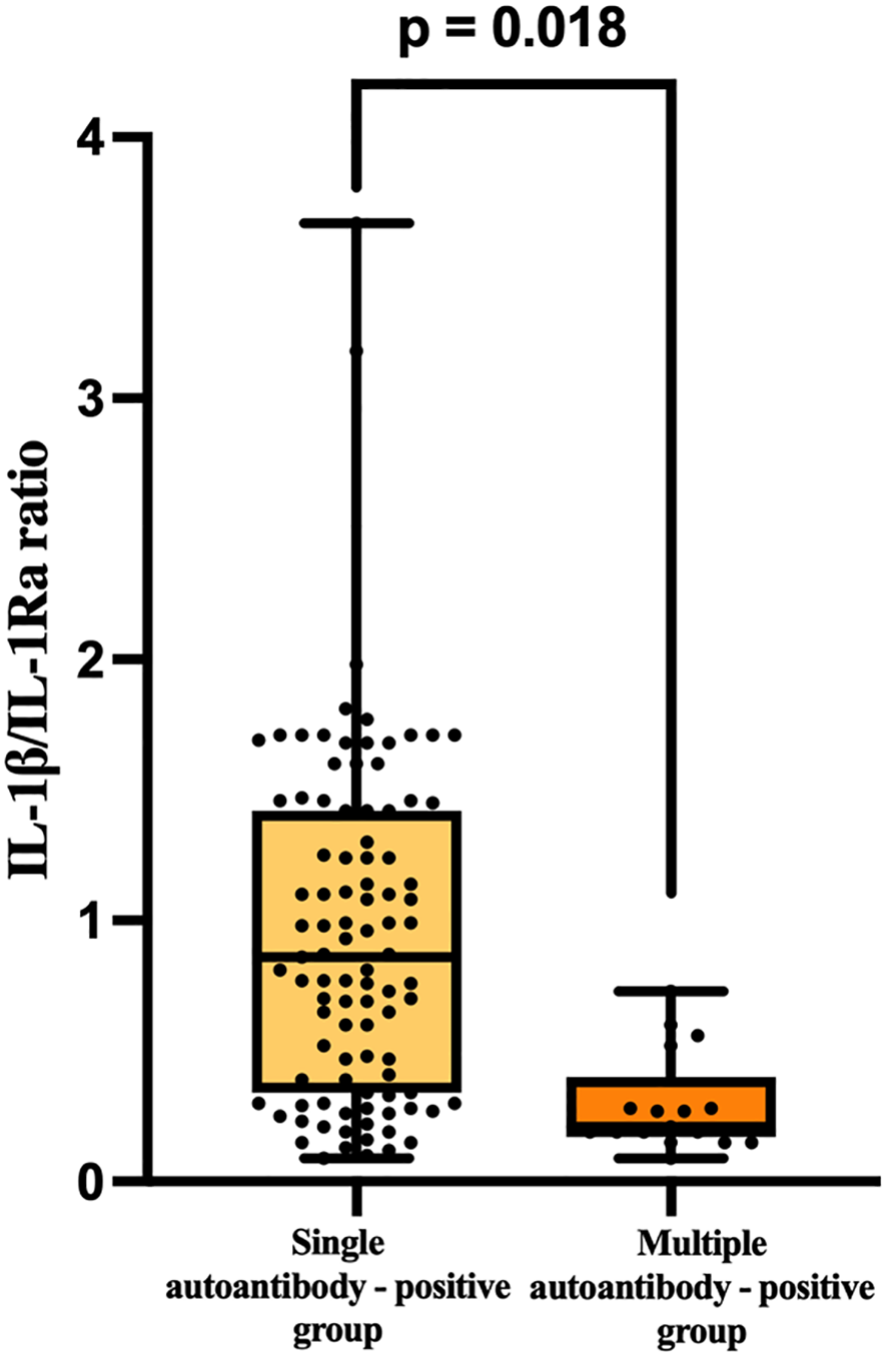
Comparison of the IL-1β/IL-1Ra ratio between single and multiple autoantibody-positive patients. Differences were assessed using the Mann–Whitney U test.

When autoantibody-positive patients were further stratified according to autoimmune burden, the IL-1β/IL-1Ra ratio differed significantly between patients with single and multiple autoantibody positivity. Patients with single autoantibody positivity showed a higher IL-1β/IL-1Ra ratio compared with those with multiple autoantibodies, suggesting that inflammatory imbalance may be more pronounced at earlier stages of autoimmune involvement (Z = −2.370, p = 0.018) (**Figure 4**).

To explore the potential role of IL-1 axis imbalance in autoantibody positivity, logistic regression analysis was performed (**Table 3**). In univariate analysis, autoantibody positivity was inversely associated with basal C-peptide levels and body mass index, and positively associated with the IL-1β/IL-1Ra ratio. Fasting insulin showed a borderline association, whereas HbA1c was not significantly associated with autoantibody positivity.

**Table 3:** Factors independently associated with autoantibody positivity.

Variable	Univariate logistic regression			Multivariate logistic regression		
	OR	95% CI	P-value	OR	95% CI	P-value
Body mass index, kg/m²	0.93	0.88-0.98	0.01			
HbA1c, %	1.04	0.95-1.16	0.34			
Fasting insulin, IU/mL	0.97	0.94-1.00	0.05	1.05	1.00-1.10	0.03
C-peptide, ng/mL	0.67	0.54-0.85	0.001	0.50	0.33-0.76	0.001
IL-1β/IL-1Ra ratio	1.73	1.03-2.91	0.03	1.94	1.10-3.44	0.024

After adjustment in the multivariate model, a higher IL-1β/IL-1Ra ratio and lower basal C-peptide levels remained independently associated with autoantibody positivity, while fasting insulin also emerged as a positive independent predictor. These findings suggest that inflammatory imbalance, impaired β-cell function, and altered insulin dynamics are associated with autoantibody positivity.

**Figure 5:**
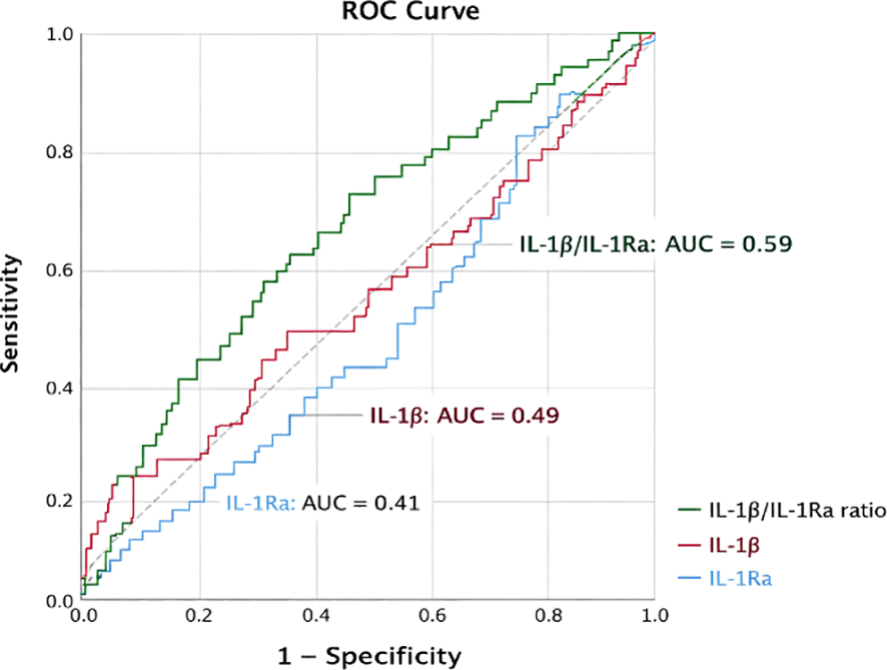
Comparative ROC curves of IL-1β, IL-1Ra, and the IL-1β/IL-1Ra ratio for identifying islet autoantibody positivity.

To assess the ability of inflammatory markers to discriminate between autoantibody-positive and autoantibody-negative patients, ROC curve analysis was performed (**Figure 5**). ROC analysis showed that IL-1Ra had a statistically significant but inverse discriminatory ability for autoantibody positivity (AUC = 0.41; 95% CI: 0.327-0.487; p = 0.029), while IL-1β demonstrated no discriminative value (AUC = 0.49; 95% CI: 0.397-0.578; p = 0.771). The IL-1β/IL-1Ra ratio showed the highest performance, with a modest but statistically significant ability to distinguish autoantibody-positive from autoantibody-negative patients (AUC = 0.59; 95% CI: 0.509–0.676; p = 0.030). The optimal cutoff value determined by the Youden index was 0.26, corresponding to a sensitivity of 69% and specificity of 42% **Table 4**. At this threshold, the positive predictive value was 32% and the negative predictive value was 78%, indicating that the IL-1β/IL-1Ra ratio may reflect inflammatory imbalance associated with islet autoantibody positivity rather than serving as a standalone diagnostic biomarker.

**Table 4:** Diagnostic performance of inflammatory biomarkers for identifying islet autoantibody positivity.

Marker	AUC	95% CI	Youden index	Optimal cutoff	Sensitivity (%)	Specificity (%)
IL-1β	0.488	0.39–0.57	0.02	36.1	79	23
IL-1Ra	0.407	0.32–0.48	0.03	36.9	90	10
IL-1β/IL-1Ra ratio	0.592	0.50–0.67	0.12	0.26	69	42

## 4 Discussion

Dysregulation of the IL-1β/IL-1Ra ratio was already evident at the time of diabetes diagnosis and differed according to islet autoantibody status. Although circulating IL-1β concentrations did not differ between autoantibody-negative and autoantibody-positive individuals, autoantibody-positive individuals exhibited significantly reduced IL-1Ra levels, indicating impaired anti-inflammatory counter-regulation. Importantly, the IL-1β/IL-1Ra ratio - rather than absolute cytokine concentrations - consistently distinguished autoantibody-positive from autoantibody-negative individuals and remained independently associated with autoantibody positivity after adjustment for metabolic parameters and β-cell function. This observation supports the concept that immune dysregulation contributes to the heterogeneity of adult-onset diabetes, extending beyond traditional classifications of type 1 and type 2 diabetes (4, 7, 10, 15). This interpretation is supported by evidence that IL-1Ra acts as a key endogenous inhibitor of IL-1 signalling, and its reduced availability can lead to unopposed IL-1 activity and amplification of inflammatory responses (16).

This pattern aligns with experimental evidence demonstrating a central role of the IL-1 axis in β-cell dysfunction. Seminal experimental and translational studies by Donath and Maedler showed that human β-cells locally produce IL-1β in response to metabolic stress, leading to impaired insulin secretion and apoptosis, while IL-1Ra acts as a critical endogenous protective mechanism (17). Notably, β-cell damage in these models occurred despite minimal systemic IL-1β elevation, highlighting the importance of local signalling balance (4, 15). Our findings are consistent with this concept, suggesting that systemic cytokine measurements may not fully capture underlying islet-level inflammatory processes. The observed reduction in circulating IL-1Ra may therefore reflect impaired compensatory mechanisms rather than increased systemic IL-1β production.

The absence of systemic IL-1β differences between groups is biologically plausible, as IL-1β primarily acts locally within pancreatic islets, adipose tissue, and the vascular compartment, whereas IL-1Ra is released into the circulation as part of a compensatory response. Consequently, circulating IL-1Ra and the IL-1β/IL-1Ra ratio may serve as more sensitive indicators of chronic low-grade IL-1 pathway activation than IL-1β alone (18). This may explain why IL-1β concentrations did not differ between groups in our study, despite clear differences in IL-1Ra levels.

Clinically, these findings highlight the heterogeneity of adult-onset diabetes. Our data suggest features consistent with a LADA-like immunometabolic phenotype rather than classical type 2 diabetes, although definitive classification requires longitudinal confirmation. The independent association between a higher IL-1β/IL-1Ra ratio and lower basal C-peptide levels further links inflammatory imbalance to reduced β-cell reserve. Importantly, the modest discriminative performance of the IL-1β/IL-1Ra ratio supports its role as an integrative marker of immune–metabolic interaction rather than a diagnostic tool for diabetes reclassification (8, 19).

Taken together, our findings suggest that early inflammatory heterogeneity in adult-onset diabetes may be associated with differences in the capacity to counter-regulate IL-1 signalling rather than the magnitude of IL-1β production. Interestingly, the IL-1β/IL-1Ra ratio exhibited distinct patterns across different autoantibody subtypes, highlighting the heterogeneity of immune-mediated processes in adult-onset diabetes. While no meaningful differences were observed in GADA-positive individuals, higher ratio values were consistently noted in ZnT8A- and IA-2A-positive groups, suggesting a tendency toward enhanced proinflammatory imbalance in these subtypes. In contrast, ICA-positive individuals demonstrated a lower ratio compared to their negative counterparts, indicating a potentially different immunopathological mechanism with less prominent involvement of the IL-1 axis. Although these differences did not reach statistical significance, likely due to limited subgroup sizes, the observed trends support the concept that the contribution of IL-1–mediated inflammation varies according to the specific autoantibody profile, reflecting underlying immunometabolic heterogeneity rather than a uniform autoimmune phenotype. Within this framework, the IL-1β/IL-1Ra ratio represents a functional marker of inflammatory regulation rather than a simple cytokine marker (20).

An important finding of our study was the non-linear pattern of IL-1β/IL-1Ra imbalance according to autoantibody burden. Single autoantibody positivity was associated with a higher IL-1β/IL-1Ra ratio, suggesting a state of insufficient anti-inflammatory compensation and heightened inflammatory imbalance. In contrast, multiple autoantibody positivity may represent a more advanced stage associated with β-cell dysfunction and altered immune responsiveness, potentially contributing to a relative attenuation of IL-1–mediated inflammatory signalling (21). Moreover, increased regulatory activity, including follicular regulatory T (Tfr) cells expressing IL-1 decoy receptors (IL-1R2) and IL-1 receptor antagonist (IL-1Ra), may further attenuate IL-1–dependent signalling despite ongoing autoimmunity (22).

## 5 Conclusion

This study demonstrates that dysregulation of the IL-1β/IL-1Ra ratio is present at the time of diabetes diagnosis and varies according to islet autoantibody status. Distinct inflammatory profiles were observed across autoantibody-defined groups, reflecting immunometabolic heterogeneity in adult-onset diabetes within the Kazakh population. These findings support the relevance of the IL-1β/IL-1Ra ratio as an integrative marker of immune–metabolic interaction in early-stage diabetes.

## 6 Limitations of the study

The cross-sectional design of this study precludes causal inference and limits the ability to assess temporal relationships between IL-1 axis dysregulation, islet autoimmunity, and β-cell function. Longitudinal studies are required to determine whether the observed IL-1β/IL-1Ra imbalance represents an early immunometabolic feature or reflects changes during disease progression.

In addition, circulating cytokine measurements may not adequately capture local IL-1 signalling within pancreatic islets or other metabolically active tissues, where IL-1–mediated effects are likely to be more biologically relevant. Islet autoantibodies were assessed at a single time point, and potential temporal changes in autoantibody profiles or titres were not evaluated. Furthermore, the relatively small number of participants with multiple autoantibody positivity may have limited statistical power for subgroup analyses.

Finally, the study was conducted in a single geographic region and predominantly included individuals of the Kazakh population, which may limit the generalizability of the findings. Despite these limitations, the study provides evidence for early immunoinflammatory heterogeneity in newly diagnosed adult-onset diabetes.

## Data Availability

The raw data supporting the conclusions of this article will be made available by the authors, without undue reservation.
